# γ-Valerolactone as Bio-Based Solvent for Nanofiltration Membrane Preparation

**DOI:** 10.3390/membranes11060418

**Published:** 2021-05-31

**Authors:** Muhammad Azam Rasool, Ivo F. J. Vankelecom

**Affiliations:** Membrane Technology Group (MTG), Division cMACS, Faculty of Bioscience Engineering, KU Leuven, Celestijnenlaan 200F, P.O. Box 2454, 3001 Leuven, Belgium; ma.rasool@kuleuven.be

**Keywords:** bio-based solvent, green solvent, polymer solubility, polymeric membranes, nanofiltration

## Abstract

γ-Valerolactone (GVL) was selected as a renewable green solvent to prepare membranes via the process of phase inversion. Water and ethanol were screened as sustainable non-solvents to prepare membranes for nanofiltration (NF). Scanning electron microscopy was applied to check the membrane morphology, while aqueous rose Bengal (RB) and magnesium sulphate (MgSO_4_) feed solutions were used to screen performance. Cellulose acetate (CA), polyimide (PI), cellulose triacetate (CTA), polyethersulfone (PES) and polysulfone (PSU) membranes were fine-tuned as materials for preparation of NF-membranes, either by selecting a suitable non-solvent for phase inversion or by increasing the polymer concentration in the casting solution. The best membranes were prepared with CTA in GVL using water as non-solvent: with increasing CTA concentration (10 wt% to 17.5 wt%) in the casting solution, permeance decreased from 15.9 to 5.5 L/m^2^·h·bar while RB rejection remained higher than 94%. The polymer solubilities in GVL were rationalized using Hansen solubility parameters, while membrane performances and morphologies were linked to viscosity measurements and cloudpoint determination of the casting solutions to better understand the kinetic and thermodynamic aspects of the phase inversion process.

## 1. Introduction

Membrane technology offers separations in the chemical industry and water treatment of small molecules (solutes) from solvent or water streams by using membranes and providing an economically viable alternative for separation and purification [1,2]. In nanofiltration (NF), the separation process is run under pressure, rejecting molecules with a molecular weight of 200–1000 Da.

Several techniques have been applied to prepare polymeric membranes, including temperature and non-solvent induced phase separation (TIPS and NIPS) [3,4,5]. Among all techniques, NIPS is the most versatile and widely used. In the NIPS process, a film cast from a polymer solution is immersed in a coagulation bath. Upon immersion, demixing occurs, resulting in the solidification of the polymer and creation of a porous structure. Controlling the process of demixing in the polymer film allows the desired membrane morphology to be fine-tuned [6,7,8,9,10,11,12,13,14,15,16,17,18]. NF membranes are asymmetric and often prepared via NIPS. Asymmetric membranes consist of a thin active separation layer on a much thicker, more open support to provide mechanical strength. NF is used for water softening, micro pollutant removal, dye removal, pretreatment for desalination and heavy metal removal.

As membrane processes are appearing increasingly in industrial applications, it is becoming more important that membrane preparation itself becomes “green” [19,20,21,22]. Membrane manufacturing currently produces over 50 billion liters of contaminated wastewater annually [21]. The European Chemicals Agency (ECHA), classified toxic solvents into a REACH list [2,23]. Tetrahydrofuran (THF), N,N-dimethylformamide (DMF), 1-methyl-2-pyrrolidone (NMP) and other conventional solvents are usually used in membrane preparation due to the good solubility of common polymers [18,24], but have all been classified as highly concerned solvents by ECHA [2,23]. Industrial use of DMF and THF is expected to be banned soon by the European Union, while NMP is on a watch list [25,26].

According to principles 5 and 7 of the 12 principles of green chemistry, safer solvents and auxiliaries and use of renewable feed stock are main aspects of green chemistry [27,28]. Not much work has been done yet in membrane preparation to implement sustainable solvents [5,29]. Some alternative solvents have been proposed to substitute DMF (by dimethyl sulfoxide) and 1,4-dioxane (by acetone) [30]. Green solvents, like methyl/ethyl lactate [31], ionic liquids [32,33,34], triethyl phosphate [35] and γ-butyrolactone [36] have been proposed to substitute conventional solvents in phase inversion.

γ-Valerolactone (GVL) is a non-toxic solvent with high boiling point (207 °C) [37,38] and has been applied in chemical processes and as flavor additive in perfumes [37,38,39]. GVL is obtained from acid hydrolysis of cellulose based biomass (wood). It is prepared from levulinic acid through a catalytic cyclization reaction via dehydration. During this reaction, an unstable intermediate is formed which is converted to GVL on hydrogenation, while levulinic acid is produced from hydroxymethylfurfural through a dehydration reaction [40].

A related lactone-based solvent, γ-butyrolactone, has already been used for NF membrane preparation via NIPS and TIPS [36,41]. In the current study, focus is laid on the application of GVL as potential bio-based, sustainable solvent for the preparation of NF-membranes, either by selecting a suitable non-solvent for phase inversion or by using high polymer concentrations in the casting solution.

## 2. Materials and Methods

### 2.1. Chemicals

Polysulfone (PSU, Udel P-1700 LCD) and polyimide (Matrimid) and were provided by Solvay (Belgium) and cellulose triacetate by Eastman (Belgium). Cellulose acetate, polyethersulfone and GVL were purchased from Sigma-Aldrich (Belgium). All polymers were dried for 24 h at 105 °C. Molecular weight (MW) and the structure of the polymers, feed solutes and solvent used in this work are given in Table 1.

### 2.2. Membrane Preparation

All polymers were dissolved in GVL at room temperature and stirred magnetically over 24 h by dissolving 10–20 wt% of the polymer (except for cellulose triacetate (CTA), where 17.5 wt% was found to be the upper concentration limit) in GVL, for details see Table 2. A wet casting thickness of 225 µm at a 1.5 m/minute speed on a polyethylene (PE)/polypropylene (PP) non-woven fabric (Novatexx 2413) impregnated with GVL was used to cast the solution. After casting, the films were instantaneously immersed in the non-solvent bath. All membranes were kept below 20 °C in distilled water until filtration.

### 2.3. Viscosity Measurements

The viscosity or rheological measurements for all polymer samples were done on a Anton Paar MCR 501 (Austria) with cone-plate geometries and evaporation blocker, as described in literature [8,10,42].

### 2.4. Cloudpoint Determination

The procedure for cloudpoint determination was adapted from literature, as described elsewhere [8,10,42] using water as non-solvent.

### 2.5. Filtrations

Filtrations were performed at 23 °C under pressures from 2 to 16 bar permitting filtrations of 16 membrane coupons simultaneous with a high-throughput filtration set-up [43,44]. Each membrane coupon had an active surface area of 0.000172 m^2^. Solutions of 35 µM rose Bengal (RB) or 16.7 mM MgSO_4_ in distilled water (H_2_O) were taken as feed (Table 1). Permeance is measured by the quantity of liquid that passes through the membrane per unit of area, time and pressure. Equation (1) is used to calculate permeance. Retention (rejection) is a dimensionless parameter, expressed in percentage (in % from 0 to 100%) with respect to feed solution. Equation (2) is used to determine retention, in which *C_F_* represents initial feed concentration and *C_P_* represents permeate concentration. RB-concentrations were measured on a Perkin-Elmer ultraviolet–visible (UV/VIS)-spectrophotometer at a wavelength of 548 nm. In the case of MgSO_4_, a Consort K620 conductometer was used to measure the concentration of permeate and feed.

(1) $$\mathrm{Permeance}\;\left(L/m^{2}\cdot h\cdot \mathrm{bar}\right)=\frac{\mathrm{Vol}\;\left[L\right]}{\mathrm{membrane}\;\mathrm{area}\;\left[m^{2}\right]\times ∆p\left[\mathrm{bar}\right]\times \mathrm{time}\left(h\right)\;}$$

(2) $$\mathrm{Retention}\;\left(R\right)=\left(1-\frac{C_{P}}{C_{F}}\right)\times 100\;\left[\%\right]$$

### 2.6. Membrane Morphology

For membrane morphology studies, membrane samples were broken in liquid nitrogen and coated with 2–5 nm gold/palladium as conductive layer using a Jeol-AFC HR-sputter coater. Images were acquired using a JEOL JSM 6010LV scanning electron microscopy (SEM).

### 2.7. Solubility Parameters

The Hansen solubility parameters (HSP) are used to describe the affinity between a polymer and GVL, as discussed earlier [39,42,45,46]. *Ra* (solubility parameter distance) was calculated using Equation (3), which is a measure for affinity between polymer (1) and solvent (2).

(3) $$R_{a}=\left[\sqrt{\;4{\left(\delta_{D2}-\delta_{D1}\right)}^{2}+{\left(\delta_{P2}-\delta_{P1}\right)}^{2\;}+{\left(\delta_{H2}-\delta_{H1}\right)}^{2}}\right]$$

Values for GVL are taken from literature [39,46,47,48]. See supporting information, for the details of HSP and RED (Relative energy difference) values (Tables S1–S5). Solubility parameters difference (Ra) of GVL and non-solvent is given in Table S6.

## 3. Results and Discussion

### 3.1. Phase Inversion Behavior of Polymer/γ-Valerolactone (GVL) Systems

#### 3.1.1. Introduction

To understand the role of polymer concentration in NIPS, both kinetic and thermodynamic aspects are studied in detail to see the effect on final membrane morphology and performance. Kinetics play a role in phase inversion via the diffusion of non-solvent into the polymer solution and of solvent out of the cast polymer film. These diffusion rates obviously depend on the molecular size and the viscosity of the polymer solution. Depending on this solvent/non-solvent exchange rate and the strength of the non-solvent (higher $Ra_{NS-P\;}or\;Ra_{S-NS}$ values) to phase-separate the polymer solution, two different types of demixing processes can be distinguished. In the case of instantaneous demixing, a membrane with a porous skin-layer, often with finger-like or pear-shaped macrovoids over the full cross-section, is generally formed, while denser membranes with a dense skin having sponge-like substructure are formed in delayed demixing [6,49].

#### 3.1.2. S-NS (Solvent and Non-Solvent) and NS-P (Non-Solvent and Polymer) Interaction Distance Parameters

**Figure 1:**
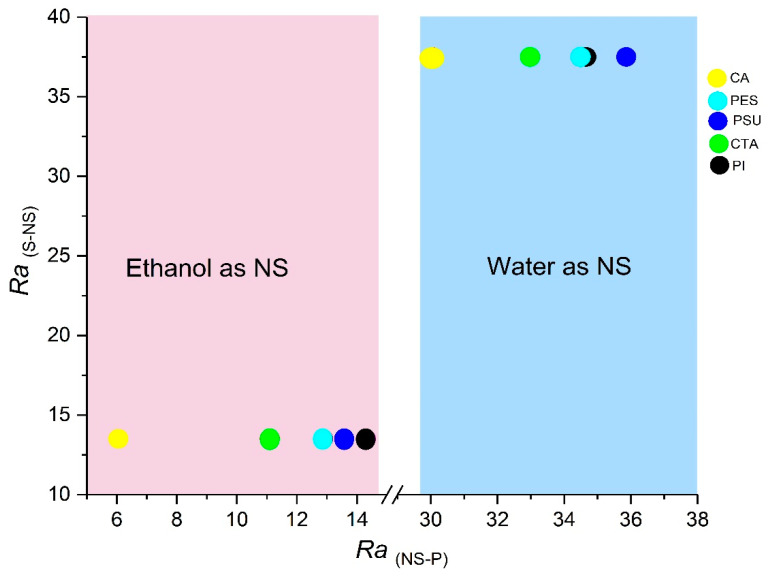
Interaction distance between solvent and non-solvent ($Ra_{S-NS})$ vs. the interaction distance between non-solvent and polymer interaction ($Ra_{NS-P\;})$.

All polymers were dissolved in GVL at room temperature with polymer concentrations from 10 wt% to 20 wt%. However, it was impossible to dissolve CTA concentrations higher than 17.5 wt%. Two different non-solvents, i.e., water and ethanol, were used in the coagulation bath to further tune the membranes toward NF-performance. To better understand the role of solvent, non-solvent and polymer in the NIPS process, the interaction distance (*Ra*) between solvent/non-solvent and between non-solvent/polymer were calculated (Table 3) and plotted in Figure 1.

Replacing water by ethanol as non-solvent increased the affinity of the polymer for the non-solvent drastically (shift to the left in Figure 1). Ethanol is thus a weaker NS and a more delayed demixing can be expected, which is supposed to lead to a more sponge-like membrane structure. In contrast, ethanol clearly has a stronger affinity for GVL (shift to the bottom in Figure 1). This should increase the driving force for NS to enter the polymer/solvent system, which would lead to more instantaneous demixing, inducing more macrovoids. These contradicting impacts of thermodynamics thus render prediction of expected membrane structures and performance very difficult.

### 3.2. Phase Diagrams

**Figure 2:**
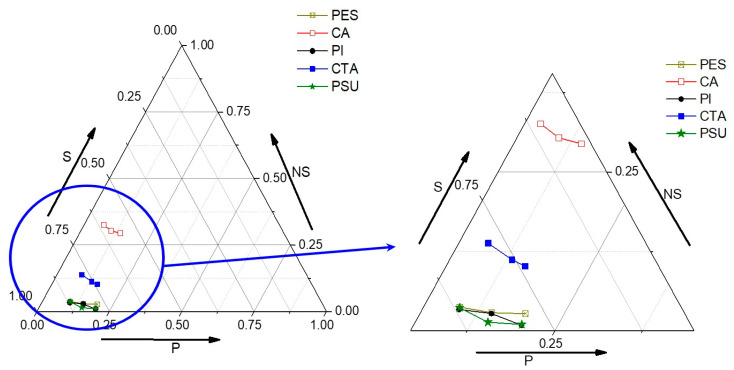
Phase diagrams of the polymer-GVL-water ternary systems obtained by the titration method to determine the cloud point (right figure = enlarged zoom).

Phase diagrams of the polymer-GVL-water ternary systems were obtained by the titration method via cloud point determination, using GVL as a solvent system and water as a non-solvent until the visual appearance of turbidity in the polymer solution (Figure 2) [6,7,50]. Cellulose acetate (CA)/GVL was the most stable system among all polymers since large amounts of water as non-solvent were required to cause turbidity [51]. The stability order of the polymer systems toward the addition of non-solvent (water) was CA > CTA > PES > PSU ≥ polyimide (PI) [52,53,54]. The $Ra_{S-P}$ values of CA, CTA, PES, PSU, and PI were above 9.0. Therefore, in theory at least, no solubility was expected. But still, these polymers were readily soluble in GVL. 

### 3.3. Kinetic Aspects of Non-Solvent Induced Phase Separation (NIPS) Process

**Figure 3:**
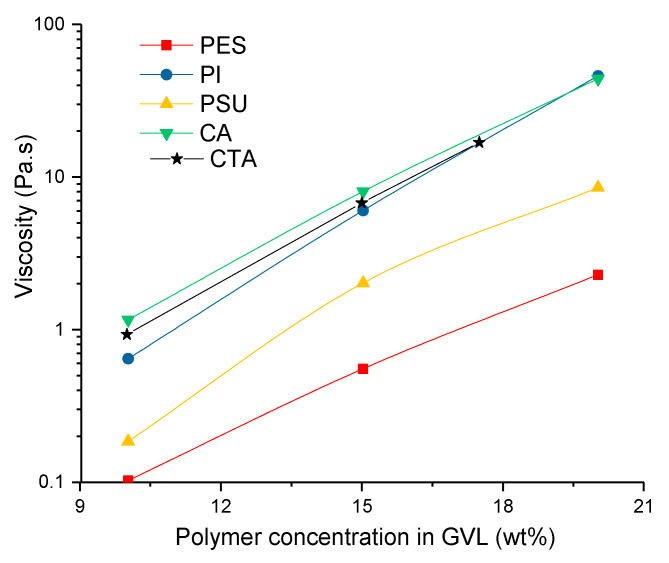
Influence of increasing polymer concentration on rheology of the casting solutions.

Kinetic aspects of NIPS can partially be understood with rheological (viscosity) measurements of the casting solutions. The viscosities of the polymer solutions logically increased with increasing polymer concentrations (Figure 3). Polymer chains entangle more in high polymer concentration solutions, and a certain minimal level of entanglement is essential to prepare sufficiently strong, defect-free membranes [55,56,57].

### 3.4. Membrane Performance and Morphology

#### 3.4.1. Influence of Cellulose Acetate (CA)

**Figure 4:**
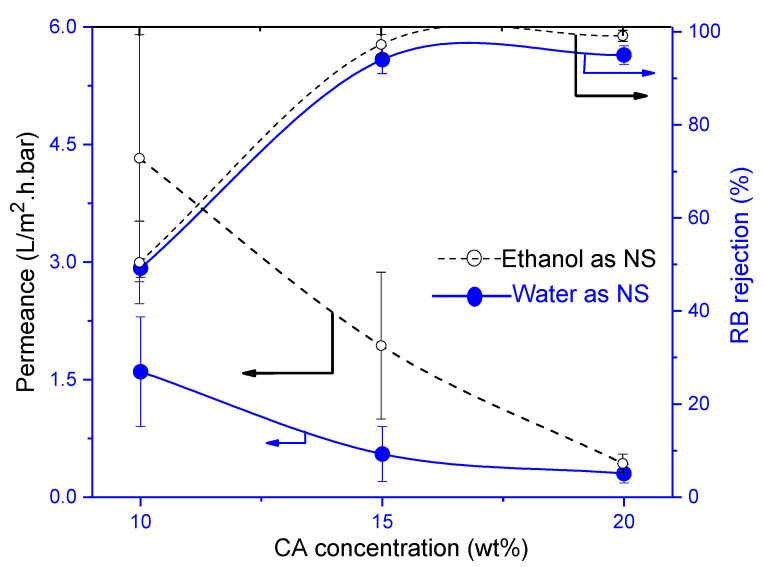
Influence of cellulose acetate (CA) concentration on membrane performance in terms of rose Bengal (RB) rejection and permeance. Striped lines represent membranes prepared using ethanol as non-solvent and full lines those with water as non-solvent.

**Figure 5:**
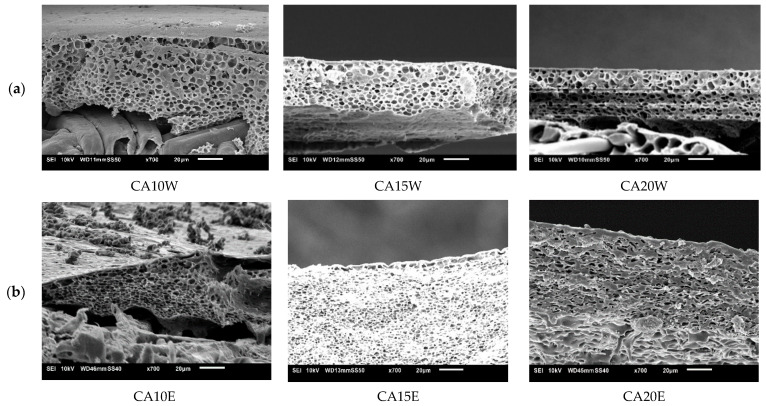
Influence of CA concentration on the scanning electron microscopy (SEM)-cross-section morphology of CA membranes cast from using (**a**) water as non-solvent and (**b**) ethanol as non-solvent.

The influence of CA concentration was noticeably seen in the permeances of the membranes. A permeance around 1.6 L/m^2^·h·bar was found for CA10W which logically decreased to 0.70 and 0.30 L/m^2^·h·bar respectively with RB rejection increasing from 49% to 95% for CA15W and CA20W membranes (Figure 4). When water was replaced with ethanol, both permeances and rejections improved. CA15E had a RB- rejection of 96% with a permeance around 2.0 L/m^2^·h·bar, qualifying very well for NF. CA20E had a lower permeance around 0.35 L/m^2^·h·bar and a slightly higher rejection of 98%. All CA membranes prepared via NIPS using either water or ethanol as non-solvent had a spongy structure (Figure 5).

#### 3.4.2. Influence of Cellulose Triacetate (CTA)

**Figure 6:**
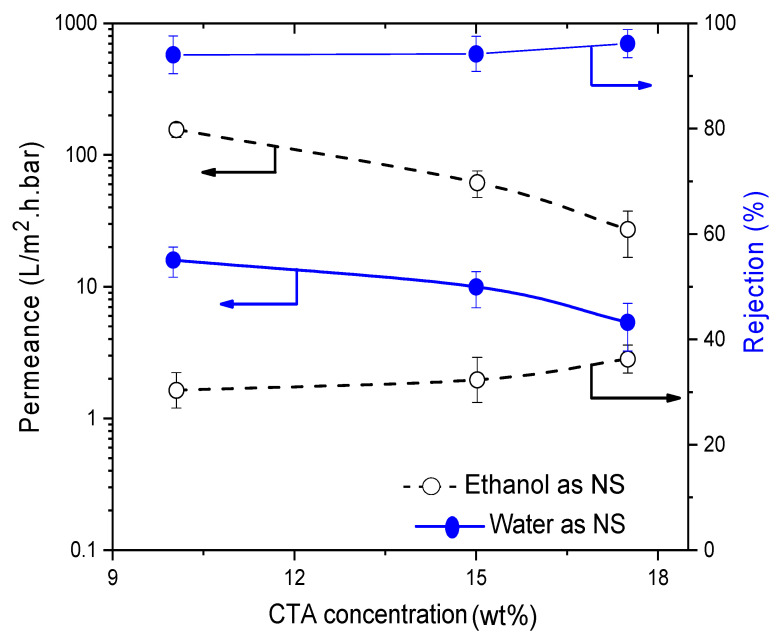
Influence of cellulose triacetate (CTA) concentration on membrane performance in terms of RB rejection and permeance. Striped lines represent membranes prepared using ethanol as non-solvent and full lines those with water as non-solvent.

As with CA, all CTA-membranes had sponge-like structures. However, also some macrovoids appeared now when using water as NS. With water as NS, RB rejection slightly increased from 94.4% to 96.8% with increasing polymer concentration but permeance decreased strongly from 15.9 to 5.5 L/m^2^·h·bar. All membranes thus had very good permeances with RB rejections over 94%, clearly suitable for NF purposes (see Figure 6).

**Figure 7:**
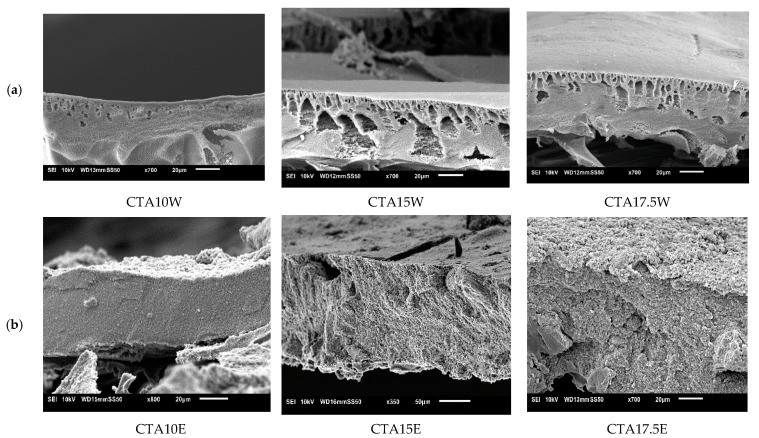
Influence of CTA concentration on SEM cross-sections of CTA membranes cast from using (**a**) water as non-solvent and (**b**) ethanol as non-solvent.

Using ethanol as NS, the spongy morphology of the CTA-membrane did not really change with increasing CTA concentration in the casting solution (see Figure 7). Permeance decreased from 158 L/m^2^·h·bar to 62 L/m^2^·h·bar on increasing CTA concentration, while RB rejection increased slightly but remained very low. These low rejections are in contract with the results of the CA-membranes.

#### 3.4.3. Influence of Polyimide (PI)

Using water as NS, PI-membrane morphology remained spongy even with changing polymer concentration. However, the influence of increasing polymer concentration was seen in membrane performance. As usual, permeances decreased from 76.5 to 10.5 L/m^2^·h·bar, while RB rejection increased from 16% to 65% but thus remained below the NF-threshold.

**Figure 8:**
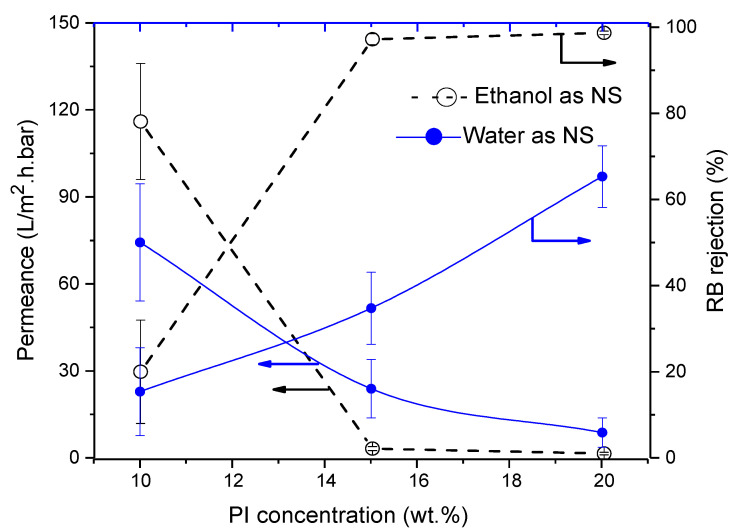
Influence of polyimide (PI) concentration on membrane performance in term of RB rejection and permeance. Striped lines represent membranes prepared using ethanol as non-solvent and full lines these with water as non-solvent.

**Figure 9:**
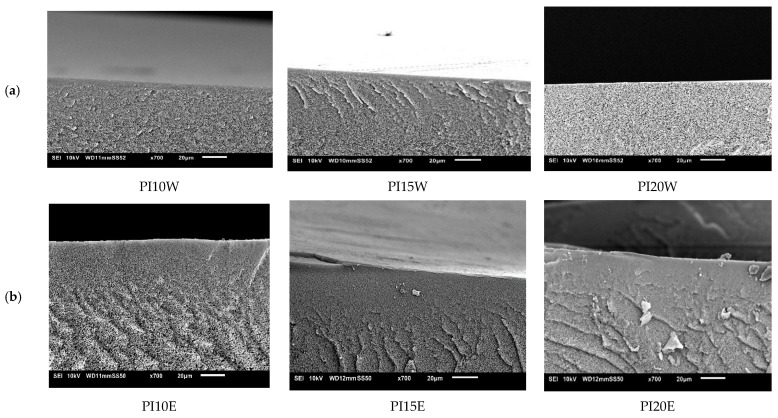
Influence of PI concentration on cross-section of the SEM images of PI membranes cast from using (**a**) water as non-solvent and (**b**) ethanol as non-solvent.

On increasing the polymer concentration, RB rejections above 97% were realized with permeances around 2.6–1.3 L/m^2^·h·bar. Although the effect of non-solvent could not be seen in membrane morphology, it is thus very visible in membrane performance (see Figure 8). When water was replaced by ethanol as NS, morphology of the PI-membranes did not change either (see Figure 9).

#### 3.4.4. Influence of Polyethersulfone (PES)

**Figure 10:**
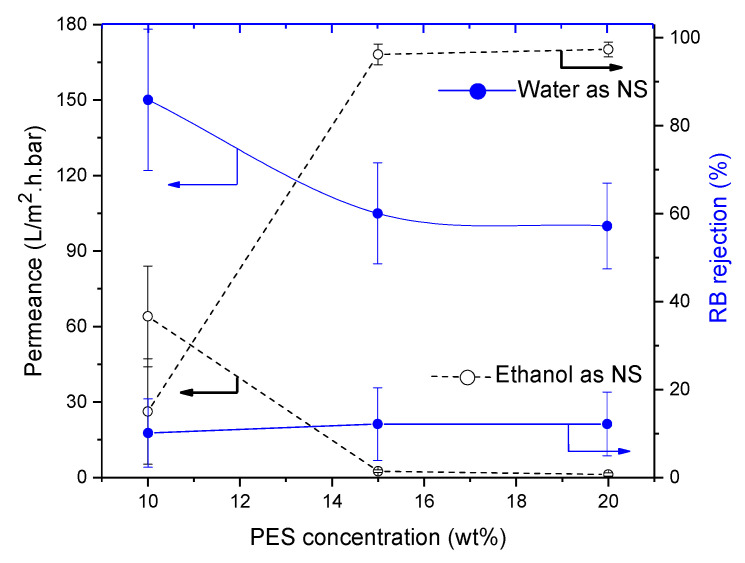
Influence of polyethersulphone (PES) concentration on membrane performance in term of RB rejection and permeance. Striped lines represent membranes prepared using ethanol as non-solvent and full lines those with water as non-solvent.

With ethanol as NS, a similar change in morphology could be observed. Due to the effect of NS, RB rejection drastically improved above 95% for PES15E and PES20E membranes, with 2.6–1.2 L/m^2^·h·bar (Figure 10) permeances. These membranes thus qualified for NF. 

**Figure 11:**
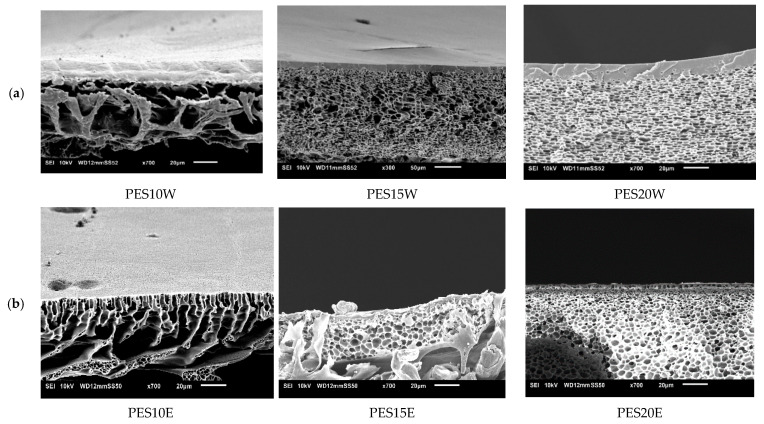
Influence of PES concentration on cross-section of the SEM images of PES membranes cast from using (**a**) water as non-solvent and (**b**) ethanol as non-solvent.

Using water as NS, a membrane with macrovoids is formed for the lowest PES concentration (Figure 11), but macrovoids totally disappeared and a sponge-like structure was formed on increasing PES concentration in the casting solution. The effect of increasing PES concentration was also seen in a permeance reductions from 151 to 100 L/m^2^·h·bar. However, RB rejection remained very low.

#### 3.4.5. Influence of Polysulfone (PSU)

**Figure 12:**
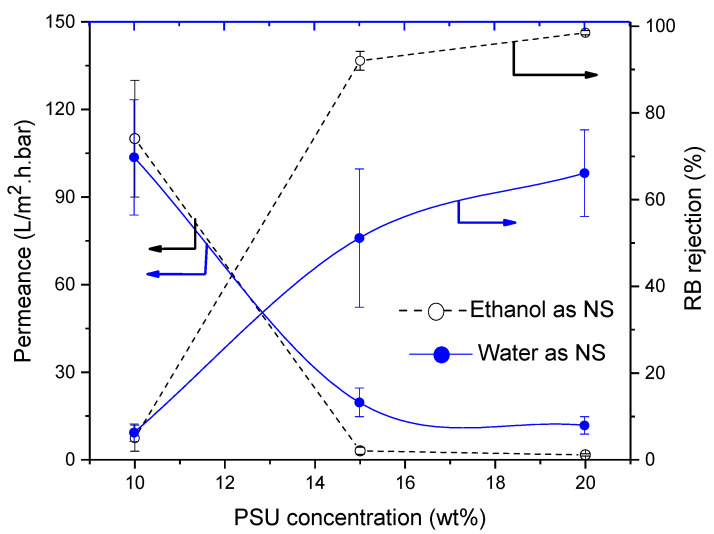
Influence of polysulphone (PSU) concentration on membrane performance in term of RB rejection and permeance Striped lines represent membranes prepared using ethanol as non-solvent and full lines those with water as non-solvent.

When water was replaced by ethanol as NS, very unusual morphologies were found for the lowest concentrations. In particular, the PSU10E membrane looked defective, as also confirmed by its performance (Figure 12). Permeances of the membranes decreased to 1.3 L/m^2^·h·bar with increasing polymer concentration in the casting solution, while RB-rejection increased to 98.5% (see Figure 12).

**Figure 13:**
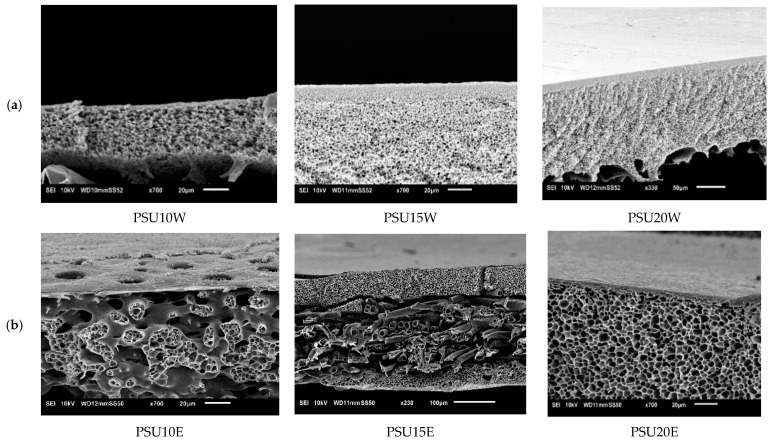
Influence of PSU concentration on cross-section of the SEM images of PSU membranes cast from using (**a**) water as non-solvent and (**b**) ethanol as non-solvent.

Using water as NS, a spongy structure was found with a quite obvious denser top layer. PES and PSU membranes are very different with respect to morphology (Figure 13). While the permeance of PSU membranes decreased from 103 to 10.5 L/m^2^·h·bar with increasing PSU concentration, RB-rejection increased from 5% to 65%, and hence was not high enough for NF.

### 3.5. Overall Comparison

**Figure 14:**
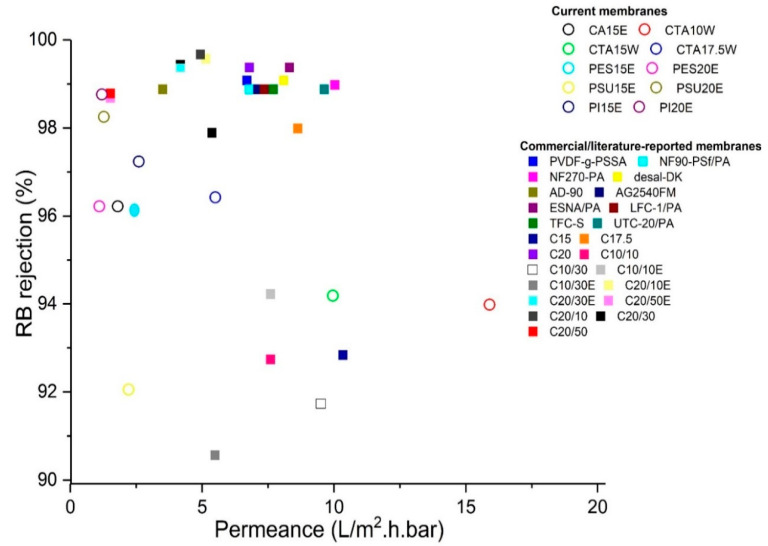
Rejection-permeance plot of nanofiltration (NF)-membranes from current work and their comparison with a selection of commercial or literature-reported membranes using a RB/H_2_O feed solution.

**Figure 15:**
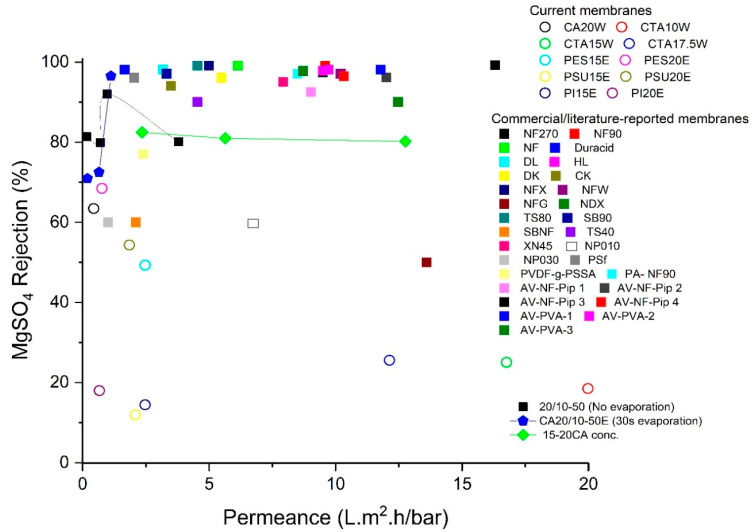
Rejection-permeance plot for membranes (which already qualified to be loose NF membranes based on the RB-data), and their comparison with a selection of commercial and literature-reported membranes using MgSO_4_/H_2_O as feed solution.

The membranes which qualified for NF (having RB rejection above 90%), were also tested using a MgSO_4_/H_2_O feed solution. None of the membranes had MgSO_4_ rejection above 90%. These membranes are thus clearly suitable for loose NF and a comparison of current membranes with a selection of commercial or membranes from literature is given in Figure 14 (permeances vs. RB-rejection) and Figure 15 (permeances vs. MgSO_4_ rejections) (for more details, see S.I. Tables S7 and S8).

When aiming for a high-permeance, CTA membranes (CTA10W, CTA 15W and CTA17.5W) are the best option (permeances ranging from 15.9 to 5.5 L/m^2^·h·bar) with all RB rejections above 90%. When selectivity is more important, PES membranes are preferred with 68% MgSO_4_ and a permeance of 1.1 L/m^2^·h·bar. Permeances and MgSO_4_ rejections are in general low as compared to commercial membranes which typically range from 1.0 to 16.3 L/m^2^·h·bar and 60.0% to 99.2% rejections [58]. The current membranes had comparable permeance, however, the MgSO_4_ rejections were lower.

CTA10W is the best membrane having a permeance around 15.9 L/m^2^·h·bar with a 94% RB-rejection among all membranes prepared from polymer/GVL systems, while PES15E is the best based on performance in terms of MgSO_4_ rejection.

When sustainability is concerned with membrane preparation, bio-based materials (i.e., CA or CTA) with sustainable solvents are always suggested over petroleum-based ones. However, there is currently a tradeoff still between selectivity and sustainability.

To combine high rejection with a high permeance seems challenging for polymer/GVL systems within the studied parameters space of polymer type, polymer concentration and NS choice. However, other parameters like, e.g., membrane annealing, coagulation bath temperature and composition, co-solvent addition in the casting solution can still be screened to further optimize these properties. γ-butyrolactone (GBL) was previously used in membranes preparation, e.g., polyvinylidene flouride (PVDF) membranes were prepared via TIPS and polyetheretherketone (PEEK-WC) membranes via NIPS. However, the use of GBL was limited to these 2 polymers. While GVL not only replaced GBL, but it also provided more opportunities to prepare NF membranes as it can be combined with common membrane polymers, like CA, PI, PES, CTA and PSU, making it an interesting solvent for membrane preparation, superior to GBL.

## 4. Conclusions

NF membranes based on CA, PI, CTA, PES and PSU have been successfully prepared using GVL as a bio-based green solvent via NIPS.

For all polymer types, the NF-criterion with RB-rejections above 90% could be fulfilled by tuning the membrane preparation using water or ethanol as non-solvent. The best membrane, CTA10W, was prepared using water as a non-solvent, from low CTA concentration. It had a permeance of 15.9 L/m^2^·h·bar and a 94% RB-rejection. Other membranes prepared from PES, PI, PSU and CA had a reasonable permeance with RB-rejection over 90%.

Loose NF membranes prepared by using polymer/GVL systems still have potential to be further tuned toward tight NF, but can obviously already serve as a ultrafiltration membrane or as a support layer in thin film composite preparation.

## Figures and Tables

**Table 1:** Molecular weight (MW), structure of polymers, feed solutes and solvent used in this work.

Polymer/Solvent	MW (kDa)	Structure
Polyimide (PI)	90–134	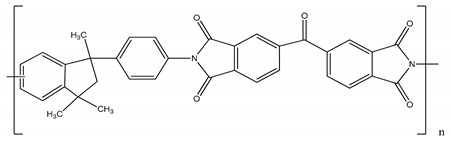
Cellulose acetate (CA)	∼30	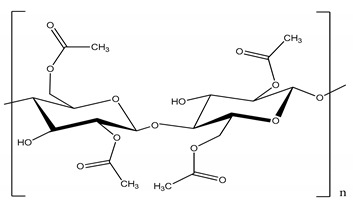
Polysulfone (PSU)	21	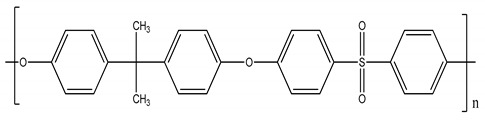
Cellulose triacetate (CTA)	278–282	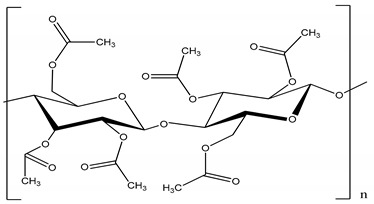
Polyethersulfone (PES)	∼35	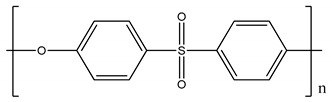
Magnesium sulfate (MgSO_4_)	0.12	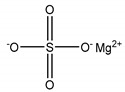
Rose Bengal (RB)	1.017	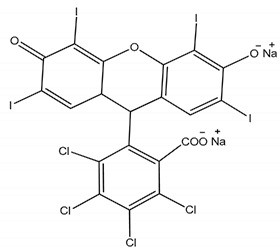
γ-Valerolactone (GVL)	0.100	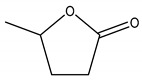

**Table 2:** Concentration of polymer in the casting solutions, the applied non-solvent and the membrane ID. γ-Valerolactone (GVL) was used as solvent for all membrane preparations.

Membrane ID	Polymer Type and Concentration	Non-Solvent
CA10W	10 wt% CA	water
CA15W	15 wt% CA	water
CA20W	20 wt% CA	water
CA10E	10 wt% CA	ethanol
CA15E	15 wt% CA	ethanol
CA20E	20 wt% CA	ethanol
CTA10W	10 wt% CTA	water
CTA15W	15 wt% CTA	water
CTA17.5W	17.5 wt% CTA	water
CTA10E	10 wt% CTA	ethanol
CTA15E	15 wt% CTA	ethanol
CTA17.5E	17.5 wt% CTA	ethanol
PI10W	10 wt% PI	water
PI15W	15 wt% PI	water
PI20W	20 wt% PI	water
PI10E	10 wt% PI	ethanol
PI15E	15 wt% PI	ethanol
PI20E	20 wt% PI	ethanol
PES10W	10 wt% PES	water
PES15W	15 wt% PES	water
PES20W	20 wt% PES	water
PES10E	10 wt% PES	ethanol
PES15E	15 wt% PES	ethanol
PES20E	20 wt% PES	ethanol
PSU10W	10 wt% PSU	water
PSU15W	15 wt% PSU	water
PSU20W	20 wt% PSU	water
PSU10E	10 wt% PSU	ethanol
PSU15E	15 wt% PSU	ethanol
PSU20E	20 wt% PSU	ethanol

**Table 3:** Solubility parameters of the polymers, GVL and non-solvents.

Polymers/Solvent	HSP Values			*Ra*(NS−P) (MPa^1/2^)		*Ra*(S−P) (MPa^1/2^)
	$\delta_{D}$ MPa^1/2^	$\delta_{P}$ MPa^1/2^	$\delta_{H}$ MPa^1/2^	H_2_O	C_2_H_5_OH	GVL
CA	18.6	12.7	11.0	30.1	6.1	11.0
CTA	18.4	11.9	10.1	32.9	11.1	9.9
PI	20.9	11.3	9.7	34.7	14.2	13.0
PSU	19.7	8.3	8.3	35.9	13.6	9.3
PES	19.6	10.8	9.2	34.5	12.9	10.5
GVL	15.5	4.7	6.6			
H_2_O	15.5	16.0	42.3			
C_2_H_5_OH	15.8	8.8	19.4			

## Data Availability

Not applicable.

## Supplementary Materials

The following are available online at https://www.mdpi.com/article/10.3390/membranes11060418/s1, Table S1: Hansen solubility parameters and relative energy difference (RED) for CA. Table S2: Hansen solubility parameters and relative energy difference (RED) for PI. Table S3: Hansen solubility parameters and relative energy difference (RED) for PSU. Table S4: Hansen solubility parameters for PES and relative energy difference (RED) for PES. Table S5: Hildebrand/Hansen solubility parameters of GVL and interaction distance for CTA. Table S6: Solubility parameters difference (Ra) of GVL and non-solvent. Table S7: Comparison of the overall membranes performance of current membranes and a selection of lab-made and commercial membranes. Table S8: Comparison of the overall membranes performance of current membranes and a selection of lab-made and commercial membranes using MgSO_4_ feed solution. References for supporting information are cited in the supplementary materials separately [1,2,3,4,5,6,7,8,9,10,11,12,13,14,15,16,17,18,19,20,21,22,23,24,25,26,27,28,29,30].
