# Supplementary material for: γ-Valerolactone as Bio-Based Solvent for Nanofiltration Membrane Preparation
Source: Membranes (Basel). 2021 May 31;11(6):418. doi: 10.3390/membranes11060418 (PMC8228422; doi:10.3390/membranes11060418)
Supplement: Supplementary file 1 [file membranes-11-00418-s001.zip › membranes-1230547-supplementary.pdf]

membranes

Supplementary Materials

γ-Valerolactone as Bio-Based Solvent for Nanofiltration Membrane Preparation

Muhammad Azam Rasool and Ivo F. J. Vankelecom *

Membrane Technology Group (MTG), Division cMACS, Faculty of Bioscience Engineering, KU Leuven, Celestijnenlaan 200F, P.O. Box 2454, 3001 Leuven, Belgium; ma.rasool@kuleuven.be
* Correspondence: ivo.vankelecom@kuleuven.be; Tel.: +32-16321594

Citation: Rasool, M.A.; Vankelecom, I.F.J. γ-Valerolactone as Bio-Based Solvent for Nanofiltration Membrane Preparation. *Membranes* **2021**, *11*, 418. https://doi.org/10.3390/membranes11060418

Academic Editor: Enrico Drioli
Received: 6 May 2021
Accepted: 14 May 2021
Published: 31 May 2021

Publisher's Note: MDPI stays neutral with regard to jurisdictional claims in published maps and institutional affiliations.

Copyright: © 2021 by the authors. Licensee MDPI, Basel, Switzerland. This article is an open access article distributed under the terms and conditions of the Creative Commons Attribution (CC BY) license (http://creativecommons.org/licenses/by/4.0/).

Hansen solubility parameters

Hansen divided the heat of vaporization energy into 3 different parts and processed as dimensional vectors which are P, D, and H and are attributed to polar forces, dispersive forces, and hydrogen bonding respectively. Hildebrand parameter is a geometric means of HSP parameters and calculated by equation 1 [1]. From table S4, the total solubility values of polymers and GVL have difference in values but still they formed a homogeneous solution, most probably due to size of GVL.

$$\delta_t = \sqrt{\delta_D^2 + \delta_P^2 + \delta_H^2} \quad (1)$$

These three HSPs are modelled as a sphere with radius R and centered at δ_D, δ_P, and δ_H) of the polymer is plotted and R_o is the critical radius of interaction of a polymer.

HSP values for polymers and GVL is given in tables (S1–S5), which were taken from literature. [2–5] Relative energy difference (RED) is equal to Ra/R, used to explain the solubility of polymers in a given solvent by a single parameter [6–8]. Smaller values of Ra represent that they can make a homogeneous solution. A single parameter to describe solvent quality and its ability to dissolve a polymer is RED (relative energy difference), which equals Ra/R.

$$\text{RED} = \frac{R_a}{R} \quad (2)$$

While Ra is interaction distance between polymer and solvent, R is the sphere of the solubility radius of the polymer. If the RED value is smaller than 1, this indicates a high affinity

between polymer and solvent, polymer should be soluble in the given solvent. When the RED value is higher than 1, indicates a low affinity between solvent and the polymer and the polymer would probably not be soluble in given solvent. Details of the HSP and RED values are given in each table (Table S1-S5). Solubility parameters difference (Ra) of GVL and non-solvent is given in table S6.

Table S1. Hansen solubility parameters and relative energy difference (RED) for CA.

**Polymer/Solvent**	δ_D	δ_P	δ_H	δ	R	
	MPa$^{1/2}$	MPa$^{1/2}$	MPa$^{1/2}$	MPa$^{1/2}$	MPa$^{1/2}$	
CA	16	7.5	13.5	22.2	8.8	
	Hansen Solubility Parameters				Ra (MPa$^{1/2}$)	RED = R/R
	δD	δP	δH	δ		
	MPa$^{1/2}$	MPa$^{1/2}$	MPa$^{1/2}$	MPa$^{1/2}$		
GVL	15.5	4.7	6.6	17.4	10.5	1.2

Table S2. Hansen solubility parameters and relative energy difference (RED) for PI.

**Polymer/Solvent**	δ_D	δ_P	δ_H	δ	R	
	MPa1/2	MPa1/2	MPa1/2	MPa1/2	MPa$^{1/2}$	
PI(Polyimide)	20.9	11.3	9.7	25.7	13.4	
Solvent	Hansen Solubility Parameters				Ra (MPa$^{1/2}$)	RED = Ra/R
	δ_D	δ_P	δ_H	δ		
	MPa$^{1/2}$	MPa$^{1/2}$	MPa$^{1/2}$	MPa$^{1/2}$		
GVL	15.5	4.7	6.6	17.4	13.1	0.9

Table S3. Hansen solubility parameters and relative energy difference (RED) for PSU.

**Polymer/Solvent**	δ_D	δ_P	δ_H	δ	R	
	MPa$^{1/2}$	MPa$^{1/2}$	MPa$^{1/2}$	MPa$^{1/2}$	MPa$^{1/2}$	
PSU	19.7	8.3	8.3	22.9	8	
Solvent	Hansen Solubility Parameters				Ra (MPa$^{1/2}$)	RED = Ra/R
	δ_D	δ_P	δ_H	δ		
	MPa$^{1/2}$	MPa$^{1/2}$	MPa$^{1/2}$	MPa$^{1/2}$		
GVL	15.5	4.68	6.56	17.4	9.4	1.1

Table S4. Hansen solubility parameters for PES and relative energy difference (RED) for PES.

**Polymer/Solvent**	δ_D	δ_P	δ_H	δ	R	
	MPa$^{1/2}$	MPa$^{1/2}$	MPa$^{1/2}$	MPa$^{1/2}$	MPa$^{1/2}$	
PES	19.6	10.8	9.2	24.2	6.2	
Solvent	Hansen Solubility Parameters				Ra (MPa$^{1/2}$)	RED = Ra/R
	δ_D	δ_P	δ_H	δ		

	MPa$^{1/2}$	MPa$^{1/2}$	MPa$^{1/2}$	MPa$^{1/2}$		
GVL	15.5	4.7	6.6	17.4	10.6	1.7

Table S5. Hildebrand/Hansen solubility parameters of GVL and interaction distance for CTA.

**Polymer/Solvent**	δ_D	δ_P	δ_H	δ	
	MPa$^{1/2}$	MPa$^{1/2}$	MPa$^{1/2}$	MPa$^{1/2}$	
CTA	18.0	12.0	10.0	23.8	
Solvent	Hansen Solubility Parameters				Ra (MPa$^{1/2}$)
	δ_D	δ_P	δ_H	δ	
	MPa$^{1/2}$	MPa$^{1/2}$	MPa$^{1/2}$	MPa$^{1/2}$	
GVL	15.5	4.7	6.6	17.4	9.5

Table S6. Solubility parameters difference (Ra) of GVL and non-solvent.

**Solvent/Non-Solvent**	δ_D	δ_P	δ_H	δ	
	MPa$^{1/2}$	MPa$^{1/2}$	MPa$^{1/2}$	MPa$^{1/2}$	
Water (as Non-Solvent)	15.5	16	42.3	47.8	
Solvent	Hansen Solubility Parameters				Ra (MPa$^{1/2}$)
	δ_D	δ_P	δ_H	δ	
	MPa$^{1/2}$	MPa$^{1/2}$	MPa$^{1/2}$	MPa$^{1/2}$	

Table S7. Comparison of the overall membranes performance of current membranes and a selection of lab-made and commercial membranes.

**Reference/ Membrane ID**	**Membrane Material**		**Permeance (L/m^{-2} h^{-1} bar^{-1})**	**Rejection (%)**		
	Support	Top Layer/Brand-Name		RB	MgSO4	NaCl
Hermans [9]	PSf	PA	2.04 (H2O)	100	96	93
Hermans [10]	XL PI	PA	2.65 (C2H5OH)	100		
Marien[11]	PSf	PA	1.23 (H2O) 0.38 (C2H5OH)	98		96
	XL PI	PA	1.09 (H2O) 0.61 (C2H5OH)	99		97
Dom [12]	XL PI	poly(β-alkanolamine)	1.99 (C2H5OH)	94		
	XL PI	poly(β-alkanolamine)	1.57 (C2H5OH)	100		
	XL PAN	poly(β-alkanolamine)	0.46 (C2H5OH)	99		
	XL PI	poly(epoxyether)	2.18 (C2H5OH)	97		
	XL PI	poly(epoxyether)	1.66 (C2H5OH)	99		
	XL PAN	poly(epoxyether)	0.47 (C2H5OH)	90		

Van Goethem [13]	PSf	PA with ZIF-8	2.70 (H2O)			82
Daem [14]		PVDF-g-PSSA	2.40 (H2O)	99	77	57
Solomon [15]	XL PI	PA	1.53 (THF)	99		
			1.54 (DMF)	99		
Solomon [16]	XL PI	polyarylate	8.0 (CH3OH)	99		
			3.8 (THF)			
Burgal [17]		PEEK	0.22(THF)			
Karan [18]	alumina	PA	52 (CH3OH)			
	XL PI	PA	19 (CH3OH)			
Evonik [11,19–21]	XL PI	Duramem 150	0.15–0.35 (C2H5OH)			
			0.09 (CH3OH)			
			0.10 (THF)			
		Duramem 200	0.16 (C2H5OH)			
		Duramem 300	0.28 (C2H5OH)			
			0.22-0.64			
		Duramem 500	0.43 (C2H5OH)			
Solsep [22,23]		NF030306	0.38 (C2H5OH)			
		Solsep3360	1.20 (C2H5OH)			
Dow [24] [25]		PA- NF90	6.67 (H2O)<br>8.54 (H2O)<br>10.38 (H2O)		>97	85-95
		PA (PIP)- NF270	11.04 (H2O)		>96	82
Hydranautics [24,26,27]	PA	ESNA	4.90 (H2O)			70
			4.38 (H2O)			
Yoon [26]	PA	LFC-1 (RO)	3.13 (H2O)			
Koch [24,28]		TFC-S	3.68 (H2O)			85
			11.00 (H2O)			
Toray [28]		UTC-20	9.17 (H2O)			99
		TMG-10PA (PIP)				
GE osmonics [29]		desal-DK	4.43 (H2O)		> 98	
			1.13 (C2H5OH)			
		AD-90	0.51 (H2O)			>99
		AG2540FM	2.79 (H2O)			>99
Rasool [7]	C15	CA	12.8 (H2O)	92.9	80.2	
	C17.5	CA	5.7 (H2O)	98.1	80.9	
	C20	CA	2.4 (H2O)	99.5	82.5	
	C10/10	CA	3.5 (H2O)	92.8		
	C10/30	CA	1.3 (H2O)	91.8		
	C10/10E	CA	3.5 (H2O)	94.3		
	C10/30E	CA	1.3 (H2O)	90.6		

	C20/10E	CA	1.1 (H2O)	99.7	96.5
	C20/30E	CA	0.7 (H2O)	99.5	71.5
	C20/50E	CA	0.2 (H2O)	98.8	63.9
	C20/10	CA	1.0 (H2O)	99.8	92.9
	C20/30	CA	0.7 (H2O)	99.7	79.9
	C20/50	CA	0.2 (H2O)	98.9	81.3
Current membranes	CA15E	CA	1.9 (H2O)	96.2	
	CTA10W	CTA	20.1 (H2O)	94.0	
	CTA15W	CTA	16.8 (H2O)	94.3	
	CTA17.5W	CTA	12.2 (H2O)	98.8	
	PES15E	PES	2.6 (H2O)	96.2	
	PES20E	PES	1.1 (H2O)	96.4	
	PSU15E	PSU	2.2 (H2O)	92.1	
	PSU20E	PSU	0.7(H2O)	98.5	
	PI15E	PI	2.5(H2O)	98.7	
	PI20E	PI	0.8 (H2O)	98.7	

Table S8. Comparison of the overall membranes performance of current membranes and a selection of lab-made and commercial membranes using $MgSO_4$ feed solution.

Manufacturer	Type	Polymer	MgSO4 Rejection (%)	Permeance ($L/m^{-2}\ h^{-1}\ bar^{-1}$)
Dow Filmtec™	NF270	Polyamide-TFC	99.2	16.3
	NF90	Polyamide-TFC	99.0	9.5
	NF	Polyamide-TFC	99.0	6.1
GE Osmonics™	Duracid	Polyamide-TFC	98.0	1.6
	DL	Polyamide-TFC	98.0	3.1
	HL	Polyamide-TFC	98.0	9.7
	DK	Polyamide-TFC	96.0	5.4
	CK	Cellulose Acetate	94.0	3.4
SynderTM	NFX	Polyamide-TFC	99.0	4.9
	NFW	Polyamide-TFC	97.0	10.2
	NFG	Polyamide-TFC	50.0	13.6
	NDX	Polyamide-TFC	90.0	12.4
	TS80	Polyamide-TFC	99.0	4.5
TriSep™	SB90	Cellulose Acetate Blend	97.0	3.3
	SBNF	Cellulose Acetate	60.0	2.1
	TS40	Polypiperazine-amide-TFC	90.0	4.5
	XN45	Polypiperazine-amide-TFC	95.0	7.9
Microdyn Nadir™	NP010	PES	60.0	5.0
	NP030	PES	60.0	1.0
S. W. Lin et al	AV-NF-Pip 1		92.5	9.0

[5]	AV-NF-Pip 2	96.1	12.0
	AV-NF-Pip 3	97.4	9.4
	AV-NF-Pip 4	96.4	10.3
	AV-PVA-1	98.0	11.7
	AV-PVA-2	97.8	9.4
	AV-PVA-3	97.7	8.7
	AV-PVA-4	96.5	8.5
	AV-NF-TMC 1	97.3	9.4
	AV-NF-TMC 2	98.2	12.0
	AV-NF-TMC 3	97.6	13.0
Rasool et al [7]	CA15	80.2	12.6
	CA17.5	80.9	5.6
	CA20	82.4	2.4
	CA20/0	80.1	3.8
	CA20/10	92.1	1.0
	CA20/30	79.9	0.7
	CA20/50	81.3	0.2
	CA20/10E	96.5	1.1
	CA20/30E	72.5	0.6
	CA20/50E	70.9	0.2
	CA20W	63.2	0.6
	CTA10W	18.5	20.1
	CTA15W	25.1	16.8
	CTA17.5W	25.5	12.2
	PES15E	49.2	2.6
	PES20E	68.1	1.1
	PSU15E	12.5	2.2
	PSU15E	54.1	0.7
	PI15E	14.5	2.6
	PI20E	18.3	0.8

Acknowledgments

Azam Rasool acknowledges and thanks to Aurora Teodorescu (Auras Dragalas from Brasov Romania) for fruitful discussion, helpful comments and visiting Leuven Belgium during this work. We acknowledge IWT-STW (Nanomexico AIO/150474/SBO), KU Leuven (C16/17/005), and the Belgian Federal Government through an I.A.P. - P.A.I. grant (IAP 7/05 FS2) for funding.

References

1. Barton, A.F.M. CRC Handbook of Solubility Parameters and Other Cohesion Parameters; Apple Academic Press, 2017;.
2. Krevelen, D.W. and Hoftyzer, P.J. *Properties of polymers: correlations with chemical structure*. Elsevier Publishing Company: Amsterdam, Netherlands, 1972
3. Pearce, E.M. Properties of polymers, their estimation and correlation with chemical structure – (2nd rev. ed.), D. W. Van Krevelen, Elsevier, Amsterdam–Oxford–New York, 1976, 620 pp. *J. Polym. Sci. Part C: Polym. Lett.* **1977**, *15*, 56, doi:10.1002/pol.1977.130150109.
4. *Summary for Policymakers*; Cambridge University Press (CUP), 2012; pp. 1–30;.
5. Rasool, M.A.; Vankelecom, I.F. Use of γ-valerolactone and glycerol derivatives as bio-based renewable solvents for membrane preparation. *Green Chem.* **2019**, *21*, 1054–1064, doi:10.1039/c8gc03652g.
6. Rasool, M.A.; Van Goethem, C.; Vankelecom, I.F. Green preparation process using methyl lactate for cellulose-acetate-based nanofiltration membranes. *Sep. Purif. Technol.* **2020**, *232*, 115903, doi:10.1016/j.seppur.2019.115903.

7. Rasool, M.A.; Pescarmona, P.P.; Vankelecom, I.F.J. Applicability of Organic Carbonates as Green Solvents for Membrane Preparation. *ACS Sustain. Chem. Eng.* **2019**, *7*, 13774–13785, doi:10.1021/acssuschemeng.9b01507.
8. Hermans, S.; Mariën, H.; Dom, E.; Bernstein, R.; Vankelecom, I.F. Simplified synthesis route for interfacially polymerized polyamide membranes. *J. Membr. Sci.* **2014**, *451*, 148–156, doi:10.1016/j.memsci.2013.10.005.
9. S. Hermans, H. Mariën, C. Van Goethem, I.F.J. Vankelecom. Recent developments in thin film (nano) composite membranes for solvent resistant nanofiltration, *Curr. Opin. in Chem. Eng.* **2015,** *8*, 45–54.
10. Mariën, H.; Vankelecom, I.F. Transformation of cross-linked polyimide UF membranes into highly permeable SRNF membranes via solvent annealing. *J. Membr. Sci.* **2017**, *541*, 205–213, doi:10.1016/j.memsci.2017.06.080.
11. E. Dom, Epoxy-based membranes for solvent resistant nanofiltration, PhD Thesis KU Leuven, Leuven, Belgium, 2017.
12. Van Goethem, C.; Verbeke, R.; Hermans, S.; Bernstein, R.; Vankelecom, I.F.J. Controlled positioning of MOFs in interfacially polymerized thin-film nanocomposites. *J. Mater. Chem. A* **2016**, *4*, 16368–16376, doi:10.1039/c6ta05175h.
13. Daems, N.; Milis, S.; Verbeke, R.; Szymczyk, A.; Pescarmona, P.P.; Vankelecom, I.F.J. High-performance membranes with full pH-stability. *RSC Adv.* **2018**, *8*, 8813–8827, doi:10.1039/c7ra13663c.
14. Solomon, M.F.J.; Bhole, Y.; Livingston, A. High flux hydrophobic membranes for organic solvent nanofiltration (OSN)—Interfacial polymerization, surface modification and solvent activation. *J. Membr. Sci.* **2013**, *434*, 193–203, doi:10.1016/j.memsci.2013.01.055.
15. Jimenez-Solomon, M.F.; Song, Q.; Jelfs, K.E.; Munoz-Ibanez, M.; Livingston, A. Polymer nanofilms with enhanced microporosity by interfacial polymerization. *Nat. Mater.* **2016**, *15*, 760–767, doi:10.1038/nmat4638.
16. Burgal, J.D.S.; Peeva, L.; Livingston, A. Towards improved membrane production: using low-toxicity solvents for the preparation of PEEK nanofiltration membranes. *Green Chem.* **2015**, *18*, 2374–2384, doi:10.1039/c5gc02546j.
17. Karan, S.; Jiang, Z.; Livingston, A.G. Sub-10 nm polyamide nanofilms with ultrafast solvent transport for molecular separation. *Sci.* **2015**, *348*, 1347–1351, doi:10.1126/science.aaa5058.
18. Darvishmanesh, S.; Firoozpour, L.; Vanneste, J.; Luis, P.; Degrève, J.; Van Der Bruggen, B. Performance of solvent resistant nanofiltration membranes for purification of residual solvent in the pharmaceutical industry: experiments and simulation. *Green Chem.* **2011**, *13*, 3476–3483, doi:10.1039/c1gc15462a.
19. Solomon, M.F.J.; Bhole, Y.; Livingston, A. High flux membranes for organic solvent nanofiltration (OSN)—Interfacial polymerization with solvent activation. *J. Membr. Sci.* **2012**, *423-424*, 371–382, doi:10.1016/j.memsci.2012.08.030.
20. Großeheilmann, J.; Büttner, H.; Kohrt, C.; Kragl, U.; Werner, T. Recycling of Phosphorus-Based Organocatalysts by Organic Solvent Nanofiltration. *ACS Sustain. Chem. Eng.* **2015**, *3*, 2817–2822, doi:10.1021/acssuschemeng.5b00734.
21. Darvishmanesh, S.; Robberecht, T.; Luis, P.; Degrève, J.; Van Der Bruggen, B. Performance of Nanofiltration Membranes for Solvent Purification in the Oil Industry. *J. Am. Oil Chem. Soc.* **2011**, *88*, 1255–1261, doi:10.1007/s11746-011-1779-y.
22. Van Der Bruggen, B.; Jansen, J.C.; Figoli, A.; Geens, J.; Boussu, K.; Drioli, E. Characteristics and Performance of a "Universal" Membrane Suitable for Gas Separation, Pervaporation, and Nanofiltration Applications. *J. Phys. Chem. B* **2006**, *110*, 13799–13803, doi:10.1021/jp0608933.
23. Lu, † Y.; Suzuki, ‡ T.; Zhang, † W.; Moore, J.S.; Mariñas‡, § B.J. Nanofiltration Membranes Based on Rigid Star Amphiphiles. *Chem. Mater.* **2007**, *19*, 3194–3204, doi:10.1021/cm070200a.
24. Suzuki, † T.; Lu, ‡ Y.; Zhang, ‡ W.; Moore, ‡ J.S.; Mariñas, † B.J. Performance Characterization of Nanofiltration Membranes Based on Rigid Star Amphiphiles. *Environ. Sci. Technol.* **2007**, *41*, 6246–6252, doi:10.1021/es070157s.
25. Yoon, J.; Amy, G.; Chung, J.; Sohn, J.; Yoon, Y. Removal of toxic ions (chromate, arsenate, and perchlorate) using reverse osmosis, nanofiltration, and ultrafiltration membranes. *Chemosphere* **2009**, *77*, 228–235, doi:10.1016/j.chemosphere.2009.07.028.
26. Boussu, K.; Van der Bruggen, B.; Volodin, A.; Van Haesendonck, C.; Delcour, J.; Van der Meeren, P.; Vandecasteele, C. Characterization of commercial nanofiltration membranes and comparison with self-made polyethersulfone membranes. *Desalination* **2006**, *191*, 245–253, doi:10.1016/j.desal.2005.07.025.
27. Xu, P.; Drewes, J.E. Viability of nanofiltration and ultra-low pressure reverse osmosis membranes for multi-beneficial use of methane produced water. *Sep. Purif. Technol.* **2006**, *52*, 67–76, doi:10.1016/j.seppur.2006.03.019.
28. Marchetti, P.; Solomon, M.F.J.; Szekely, G.; Livingston, A. Molecular Separation with Organic Solvent Nanofiltration: A Critical Review. *Chem. Rev.* **2014**, *114*, 10735–10806, doi:10.1021/cr500006j.
29. Lin, S.W.; Martínez-Ayala, A.V.; Pérez-Sicairos, S.; Félix-Navarro, R.M. Preparation and characterization of low-pressure and high MgSO4 rejection thin-film composite NF membranes via interfacial polymerization process. *Polym. Bull.* **2019**, *76*, 5619–5632, doi:10.1007/s00289-018-2665-7.
